# Of the Causes, Nature, and Treatment of Palsy and Apoplexy: Of the Forms, Seats, Complications and Morbid Relations of Paralytic and Apoplectic Diseases

**Published:** 1850-11

**Authors:** 


*Of the Causes, Nature, and Treatment of Palsy and Apoplexy: of the Forms, Seats, Complications and Morbid Relations of Paralytic and Apoplectic Diseases.* By JAMES COPLAND, M. D., F. R. S, etc., etc., etc., etc., etc. Philadelphia: Lea & Blanchard. 1850. 12 mo., pp. 326.

This monograph is chiefly a re-publication of the articles on palsy and apoplexy contained in the Medical Dictionary by the author. It contains a digest of facts and opinions relating to the important diseases of which it treats. For sale by Phinney & Co.

In addition to the foregoing, several new publications have been received, which will be noticed in our next number.

